# The General Medical Council's Problems

**Published:** 1918-06-08

**Authors:** 


### WAR INFLUENCES ON THE MEDICAL PROFESSION.

### The General Medical Council's Problems.

The recent session of the General Medical Council has included more subjects of general interest than are usually to be found in the proceedings of this dignified organisation. For the most part the activities of the Council seem, at least to the outsider, to be formal and remote rather than vital and immediate, and a scanty paragraph here and there is the sum of the recognition they receive from the press. To the initiated, on the other hand, the Council is known to carry considerable responsibilities and to stand on watch over matters of large concern to the public interest. But, let it be admitted, these duties have a technical quality that can hardly engage popular concern. Were they neglected troubles of various kinds would become manifest. But their performance is of the silent sort, and its effects occur in the shape of benefits that are taken as natural and proper in the life of the community. Hence, whilst failure would cause complaint, the discharge of the task does not excite gratitude, and, indeed, hardly receives attention. So much for normal times in the national life.

The Claims of the Army.

Unfortunately the times are not normal, and while all ranks and classes and professions feel the strain and disturbance of the war these have had a special incidence on the medical profession, and their influence is an increasing quantity. At first the question was the need of supplying doctors for the new armies in camp and in the field, and gradually thousands of practitioners left their civilian work to take on military duties. The general public had to accommodate themselves to the situation; and, though no doubt at the cost of individual hardships, they accepted the necessity. But still the cry of the Army for more doctors persisted, and hence, in the latest Military Service Act, power was taken to claim by conscription the services of medical men under fifty-six years of age, though the general claim of conscription did not extend beyond fifty-one years. On the doctors, therefore, has been established a bond in excess of that imposed on any other class or profession. The object, undoubtedly, is to get more doctors for the Army by regulating the distribution of men in civil practice. At present there are places where the civilian work is being carried on by relatively young men. But these men cannot be taken for military service because their removal would leave the district in which they practise destitute of all medical help. On the other hand, there are districts, principally of the residential order, where the supply of medical practitioners is in excess of the needs of the population. The new legislation, undoubtedly, finds its motive in these facts. To any practitioner under fifty-six years of age the authorities may now say "Either you must join the Army yourself, or, to gain exemption from this claim, you must be willing to work in such a fashion, or at such a place, as will set free a younger man." Thus, apart from the conspicuous character of its work in relation to the war, the medical profession is displayed to the public eye as the one class of the community which is subjected to com-

**General Medical Council's Problems—*(continued)*.**

pulsory service on what may be called the higher scale.

INDUSTRIAL CONSCRIPTION.

It is little wonder that in these circumstances the activities of the General Medical Council take on a peculiar interest. Its constituency, for so the medical profession may be named, has been the subject of special legislation, and this legislation may be justly described as industrial conscription. It is almost impossible to think of any other class in the community being placed in a similar position. As was inevitable, the President of the Council dealt with this subject in his opening address, and he suggested that the arrangement might perhaps be regarded, as a tribute to the "general standard of healthy vigour of the profession," and that the wise policy was to accept the claim as a testimonial to the unique quality of the service that the profession is capable of rendering to the State. On the other hand, voices have been heard urging the profession not to accept with meekness this exceptional demand, which is described by the protesters as a "slap in the face" and so on. One disgruntled individual has gone so far as to announce that though the Government might order him to go here or to go there they could not compel him to work, even when he was transferred to a new situation.

NOT COMPLIMENT OR TYRANNY, BUT NEED.

In all this, it seems to us, there is a degree of exaggeration both on one side and on the other. Undoubtedly to make a special class of the community the subjects of industrial conscription while leaving others of like age free from the bond is a procedure incapable of theoretical defence. Argued *in vacuo* the proposition has not a leg to stand on. Almost with equal confidence it may be affirmed that in advancing this demand the Government has no special desire to "honour" the person or calling of the medical practitioner. What rules the position is neither compliment nor tyranny, but need. Our armies must have doctors; so, also, must our civilian population. Further, the supply of doctors is a limited quantity and cannot be increased by any urgent or available means. Thus it is a vital need of the community that in so far as the doctors exist they should be distributed to the best advantage, and this as an obvious interpretation implies that the younger men should take service with the Army and the older men should replace them on Home service. But this adjustment is not possible unless powers are taken to co-ordinate the Home arrangements in such a fashion as to secure that each man shall be told his field of duty, not by his own judgment and convenience, but by those who survey the whole field of need. A full severity of criticism may easily be invoked against this plan. But what the critics must do if they wish to be effective is to show an alternative. To discuss whether the proposal is an honour or an insult is an idle enterprise. The sole question is—Is it necessary? The welfare of our soldiers and the health of our Home people are both at stake. Here is machinery which has at least the promise of efficiency, and until an equally hopeful scheme can be advanced the new legislation will hold the field.

THE DECLINE IN NUMBERS.

Whatever be the pressure of the moment, the General Medical Council, in view of its responsibilities, cannot avoid an anxious eye on the future. At present the war shows little prospect of any abatement of its imperious demands, and, whether peace or war, some forty millions of people in these islands need for their protection an adequate supply of medical practitioners; and, presumably, with an increase of population there ought to be an increase of doctors. What is the official record of the present position in this respect? The figures of the current Medical Register show a net increase of 338 in the number of registered practitioners, the increase in the previous year having been 256. But these numbers are certainly fewer than what used to be regarded as necessary to meet the wants of the normal increase of the population, so that relative to the total population there is a decrease in the medical supply. Further, directly or indirectly, as a consequence of the war, the losses by death and by disablement are increased, so that the relative diminution just mentioned is accentuated. A summary of the position is found in the statement that the new names registered in 1917 numbered 1,134, while the corresponding figure for 1914 was 1,433; that is, in 1917 300 fewer practitioners entered the profession than in the year which, in this respect, was practically undisturbed by the influence of the war. The losses by death cannot at present be confidently stated, but it is certain that a much heavier toll has been taken than in normal times. In short, there is a substantial shrinking in the medical profession in spite of the increasing numbers of women who join it, and these latter, with all the good will in the world, cannot be regarded as exactly equivalent to their male *confrères*. There are situations and difficulties which at least the average woman practitioner cannot meet, and to this extent the declining numbers of the men cannot be regarded as fully compensated by an increasing number of women. Further, it must be remembered, in this aspect of the discussion, that a larger proportion of women than of men practitioners retire from practice on marriage or under other influences. Hence, looked at broadly, a substantial decline in the total number of practitioners must be admitted, and this in the face of a population which is ever increasing.

PROSPECTS OF THE NEAR FUTURE.

Next comes the question of medical students as throwing light on the prospects of the near future. Here there is some relief in finding that the figures seem to show a decided upward tendency. The total registrations for 1917 number 2,165, which is a larger figure than in any year since 1891. But, of course, these figures can have no practical effect on the number of practitioners until 1922, this being the earliest date at which a student who commenced his curriculum in 1917 can complete his studies and add his name to the professional roll.

**General Medical Council's Problems—(*continued*).**

For 1918 and 1919 there is certain to be a serious decline in the numbers of those qualifying for practice, though a confident estimate on this point is not at present possible. On the suggestion of the President of the Medical Council the Ministry of National Service has arranged to take a census of medical students, and from this will be learned the numbers in each year of study, and thus an approximate estimate of the number of practitioners in the early future will be available. Related to this discussion is a further matter to which the President of the Council drew the attention of his audience. It is that the influx of new students includes an exceptional proportion of those under eighteen years of age. This experience raises two considerations. First it suggests that lads are being prematurely withdrawn from school to the detriment in greater or less measure of their general education, and in this way a practical test may be applied to an educational controversy of some moment. It has long been urged that the minimum standard of the preliminary examination for admission as a medical student is not sufficient to guarantee the capacity to profit by a medical training, at least in the case of the younger candidates. Here and now, it would seem, the issue is to be put to the test, and from it there is likely to be produced a decisive argument on one side or the other. In the second place, it is doubtful how far this rush of junior candidates into the ranks of the medical students can prove efficacious relative to the supply of medical practitioners. At present they are secure; but what if the war lasts until they reach the age of eighteen and so come within the grasp of the Military Service Acts? In the early days of the struggle the prospect of a four years' war seemed almost fantastic, and the military mind had no scruple in accepting, and later in demanding, medical students for the combatant ranks. The fruit of that judgment we are likely to gather in the years immediately in front of us. Is there any guarantee that the experience will not be repeated? In other words, can we be sure that out of the increased number of students a considerable proportion will not in the early future be claimed for ordinary military service? Unless this question can be answered in the negative the present figures may prove to be misleading and illusory.

### The Council and Judicial Findings.

One incident in the judicial activities of the Council during the present session is especially noteworthy. It occurred in connection with the conviction of a medical practitioner on a charge of attempting to produce a disease in a man who wished to avoid military service. The charges—there were two separate incidents—were found proven in the police court, and the practitioner was sentenced to six months' imprisonment with hard labour; the convictions were upheld on appeal to Quarter Sessions, but the sentence was reduced to six months' imprisonment in the second division. Now, it appears, that while the Council is legally bound to accept as "correct" a conviction recorded in the courts of law, and is thus restrained from appearing to act as a court of appeal, it may hear evidence and argument bearing on the gravity of the offence or may consider excuses urged in extenuation of the crime. In a word, while the Council may not review the judicial finding, it is not bound on the basis of this finding to penalise the practitioner, but may "see fit" either to remove his name from the roll or to abstain from doing so. Thus on the present occasion the Council heard evidence from the incriminated practitioner and from other practitioners who are firmly convinced of his innocence. The result was that the Council did not "see fit" to direct the Registrar to remove the name of the practitioner in question from the Medical Register. Here, then, is the position. A practitioner charged with using his professional knowledge and skill for the purpose of defeating within the measure of his action an Act of Parliament directed to the promotion of recruiting, is found guilty and sentenced to imprisonment by a civil court. When the matter comes before what may be called the professional court, where, presumably, the offence, if committed, must seem specially unworthy, the practitioner suffers no penalties and is allowed to retain his status.

### An Unsatisfactory Position.

The contrast is striking, and it is all the more impressive seeing that in another case of not dissimilar character, and heard during the same session of the Council, the convicted practitioner suffered the penalty of expulsion from the official list. The state of matters here revealed, it must be admitted, is most unsatisfactory. It is well known that the practitioner in the case here discussed has from the outset and consistently asserted his innocence of the offence with which he was charged, and his account of the circumstances has convinced not a few that he is the victim of a judicial mistake, if not of a more sinister enterprise. His retained status after the question has been debated by the General Medical Council will certainly be quoted, and reasonably quoted, in support of this view. The Council, quite rightly, is not tender to persons who use their professional opportunities and knowledge for unworthy ends, and the alleged conduct in the case here in question was unworthy and shameful in the highest degree. But having heard the discussion the Council declines to act. Presumably the opportunities for legal appeal have been exhausted by reference to the Quarter Sessions, and there only remains the clemency of the Crown under the direction of the Home Secretary. A petition to secure this is being promoted, and in view of the action, or rather inaction, of the Medical Council this is surely likely to be widely signed. A practitioner who might reasonably have hesitated prior to the hearing of the case by the Council will hardly now withhold his support. If the offence were committed, it is deserving of the maximum penalties. If, on the other hand, it cannot be supported by evidence which invokes these, the offence has no sufficient existence to demand any penalty at all.

